# Problem Management Plus (PM+) in the management of common mental disorders in a specialized mental healthcare facility in Pakistan; study protocol for a randomized controlled trial

**DOI:** 10.1186/s13033-017-0147-1

**Published:** 2017-06-08

**Authors:** Syed Usman Hamdani, Zainab Ahmed, Marit Sijbrandij, Huma Nazir, Aqsa Masood, Parveen Akhtar, Hania Amin, Richard A. Bryant, Katie Dawson, Mark van Ommeren, Atif Rahman, Fareed Aslam Minhas

**Affiliations:** 1 Institute of Psychiatry, WHO Collaborating Centre for Mental Health Research and Training, Benazir Bhutto Hospital, Rawalpindi, Pakistan; 2 Human Development Research Foundation, Islamabad, Pakistan; 3 Department of Clinical Psychology, VU University, Amsterdam, The Netherlands; 4 School of Psychology, University of New South Wales, Sydney, Australia; 5 World Health Organization (WHO), Department of Mental Health and Substance Abuse, Geneva, Switzerland; 6 University of Liverpool, Liverpool, UK

**Keywords:** Low resource settings, Psychotherapy, Low and middle income countries, Cognitive behavioural therapy, Task-shifting, Non-specialist counsellors, Common mental disorders, Depression, Anxiety, Trans-diagnostic, mhGAP, Scale-up, Integration

## Abstract

**Background:**

The World Health Organization (WHO) has developed Problem Management Plus (PM+), a 5-session, psychological intervention program delivered by trained non-specialist that addresses common mental disorders. The objectives of this study are to evaluate effectiveness and cost-effectiveness of PM+ in a specialized mental health care facility in Pakistan.

**Methods:**

A single blind individual randomized controlled trial (RCT) will be carried out in the outpatient department of a specialized mental healthcare facility in Rawalpindi, Pakistan. After informed consent, patients with high psychological distress (General Health Questionnaire-12 (score >2) and functional impairment (WHO Disability Assessment Schedule 2.0 score >16) will be randomised to PM+ plus treatment as usual (n = 96) or TAU only (n = 96). The primary outcome is the psychological distress, measured by levels of anxiety and depression on the Hospital Anxiety and Depression Scale and improvement in functioning as measured by WHODAS at 20 weeks after baseline. Secondary outcomes include improvement in symptoms of depression, post-traumatic stress disorder, levels of social support and cost effectiveness evaluation. Qualitative interviews will be conducted to evaluate the process of implementing PM+ including barriers and facilitators in implementation and possibility of integration of PM+ program in specialized mental health care facilities in Pakistan.

**Discussion:**

The results of this study will be helpful in evaluating the effectiveness of the approach of training non specialists, based in the specialized mental health care facilities in delivering evidence based psychological interventions in the low resource settings.

*Trial registration* Australian New Zealand Clinical Trials Registry, ACTRN12616000381482. Registered Retrospectively on March 23, 2016

**Electronic supplementary material:**

The online version of this article (doi:10.1186/s13033-017-0147-1) contains supplementary material, which is available to authorized users.

## Background

Promoting mental health and wellbeing universally and equitably is a key undertaking in the global sustainable development agenda. Access to care for mental health has been included as one of the indicators in the sustainable development goals [1, 2]. The treatment gap for common mental disorders in low middle income countries (LMICs) such as Pakistan is documented to be nearly 90% [3–5]. Whatever care is available, is concentrated in few urban specialized healthcare facilities which largely consists of pharmacotherapy and unstructured psychotherapy, if at all available [6]. With scarcity of mental health specialists (psychiatrists and professionally trained and certified clinical psychologists),lack of manualized, evidence based, and culturally appropriate psychological interventions, it is not possible to provide care to a large proportion of patients who present with common mental disorders in the healthcare facilities [7, 8]. WHO mhGAP programme makes available evidence-based psychological interventions for priority mental health conditions to be delivered by non-specialists in primary healthcare settings. mhGAP program implementation is ongoing in Pakistan. The specialized mental health facilities have a key role in bridging the treatment gap through the implementation of mhGAP programme in the Primary Health Care (PHC) system and by providing training, supervision and ongoing support to the non-specialists [8].

For the program to be sustainable and to bridge the treatment gap at scale, a trained workforce is needed in the specialized healthcare centres to supervise and support the delivery of psychological interventions in the primary health care settings and to deal with the ever-growing burden of patients presenting with common mental disorders in the outpatients departments of specialized facilities. Cascaded training and supervision system supported by task shifting strategies (wherein less qualified mental healthcare staff such as counsellors, social workers, health workers provide psychological interventions to the patients under specialist supervision [4, 9]) has been shown to be effective in bridging gap for mental health in low resource settings [10, 11].

This study addresses the issue of making available evidence-based psychological support by evaluating the effectiveness and cost-effectives of the implementing WHO Problem Management Plus (PM+) program in a specialized health care facility in Pakistan. PM+ is a trans-diagnostic, low intensity psychological intervention for common mental disorders (e.g. depression, anxiety, stress), developed by WHO as part of its mhGAP Program.

Problem Management Plus has following four core features:

1. It is brief; consisting of five individual sessions
2. It can be delivered by specialists (psychiatrists, psychologists) as well as trained non-specialists including social workers, health workers and volunteers
3. The intervention is trans diagnostic-addressing depression, anxiety and stress and
4. It has been designed for use in low resource settings and especially those affected by chronic adversity (e.g. violence and humanitarian crises).

Problem Management Plus consists of problem-solving and behaviour therapies techniques (problem solving counselling plus stress management, behavioural activation and social support) that are empirically supported and formally recommended by the WHO [12–15].

Problem Management Plus has been culturally adapted for use in Pakistan (Chiumento et al., forthcoming) and tested for effectiveness in the primary healthcare and community settings of Pakistan where it was found effective in reducing symptoms of anxiety and depression [16, 17].

The proposed study is aimed at evaluating the effectiveness and cost-effectiveness of a psychological intervention delivered by non-specialists based in the specialized mental health care facility of a resource poor setting, under the supervision of specialists.

## Methods

### Objectives and hypotheses

The overall aim of this randomized controlled trial (RCT) is to evaluate the effectiveness and cost-effectiveness of PM+ plus Treatment-as-Usual (TAU) compared to TAU only in the management of common mental disorders in a specialized mental healthcare facility in Rawalpindi, Pakistan.

The primary hypothesis is that PM+ plus treatment as usual is superior to TAU alone in reducing psychological distress (depression and anxiety) and improving functioning at 20 weeks post assessment. Secondary hypotheses are that:

1. Participants in the intervention arm will report reduced symptoms of depression, post-traumatic stress and PM+ intervention will result in improved levels of social support assessed at post-assessment at 7 weeks, and follow-up end-point assessments at 20 weeks.
2. PM+ plus TAU is more cost effective than TAU alone in the management of common mental disorders in a specialized mental health care facility.

**Fig. 1:**
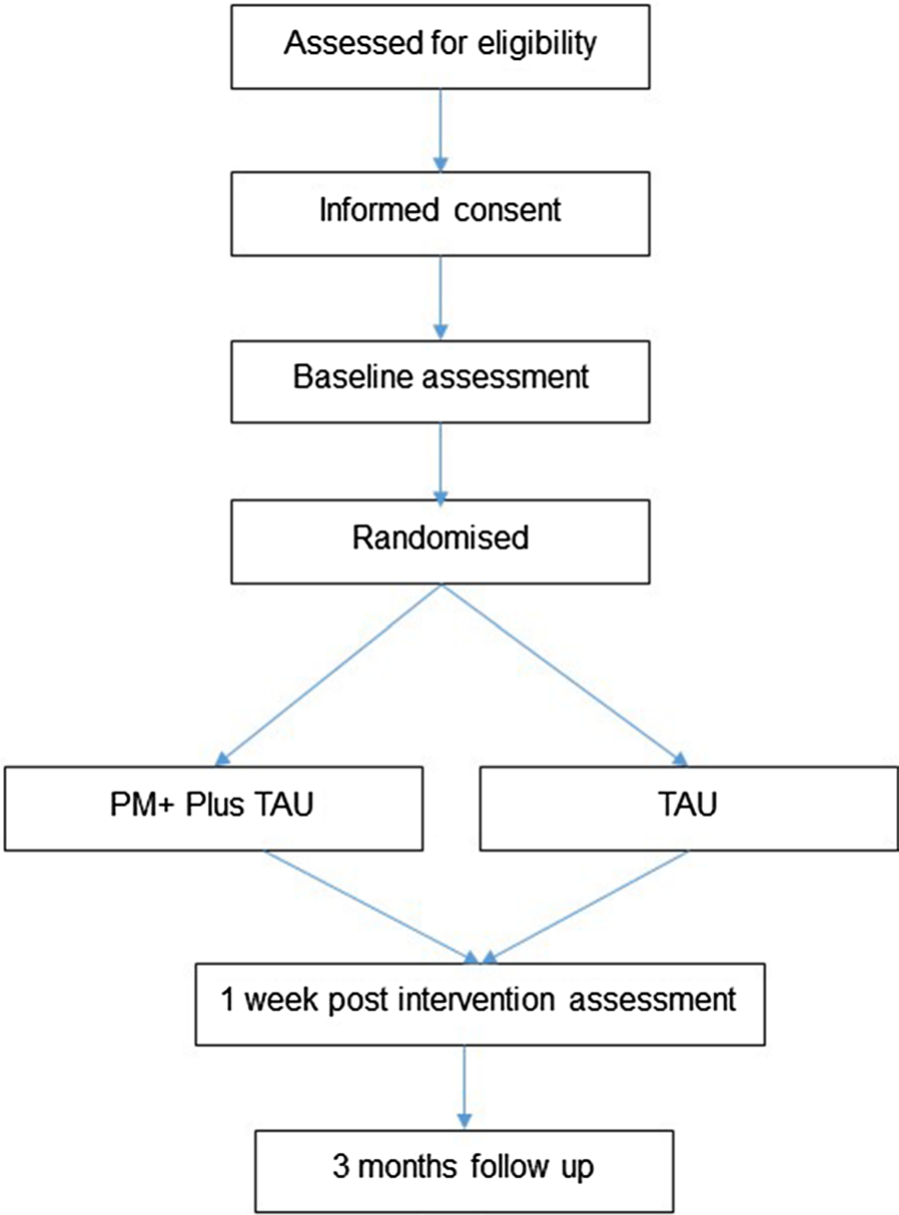
Flow diagram

Figure 1 presents an overview of the design. SPIRIT recommendations have been followed in preparing the trial protocol (Additional file 1: Appendix A).

A qualitative component will explore the feasibility of setting up specialist PM+ clinics at specialized care facilities in a LMIC such as Pakistan.

#### Setting and participants

The study will be carried out in the outpatient department of a specialized mental health care facility in Rawalpindi, Pakistan. As the only tertiary mental health care facility, it caters to the mental health care needs of the population living in the northwest of Pakistan. It is affiliated with the medical school and universities in the city and provides undergraduate and postgraduate mental health research and training to both local and international students.

The study will be conducted with adult walk-in clients presenting with common mental disorders such as depression, stress and anxiety related conditions. Inclusion criteria are: (a) adult (age 18–60 years) outpatient department attendees, referred for psychotherapy for depression, anxiety and stress related conditions by the specialists after routine evaluation; (b) score above 2 on a screening questionnaire for psychological distress (General Health Questionnaire-12; GHQ-12; [18, 19]) and (c) score above 16 on a screening questionnaire for functional impairments (WHO Disability Assessment Schedule 2.0; WHODAS; [20]). These instruments are described below. Exclusion criteria are: (a) acute medical conditions; (b) imminent suicide risk; (c), psychosis, alcohol or drug-use dependence; and (d) cognitive impairment (e.g. severe intellectual disability, dementia).

#### Procedure

Individuals who present at the out-patients department will be evaluated for depression, anxiety, or other stress related mental health conditions by a psychiatrist. After clinical evaluation, if the psychiatrist decides psychotherapy/psychosocial support is indicated, s/he will introduce the PM+ study. If the person agrees to participate in the study, the participant will be referred to the study coordinator.

The study coordinator will seek written informed consent for participation in the study. The informed consent procedure consists of two-steps: (1) Informed consent for screening and (2) Informed consent for the PM+ trial. The latter is only required for participants meeting inclusion criteria. For each step respondents who decide to participate will be asked to complete a written consent form. Each individual will be given at least 24 h to decide to take part in the trial. Illiterate participants will be asked witnessed oral consent and a thumb print in lieu of a signature, in line with recommendations from WHO [21]. The witness will not be a member of the research team.

Next, the independent assessment team, trained in the ethical conduct of research and assessments, will conduct the screening and administer the GHQ-12 and WHODAS. Participants meeting inclusion criteria (GHQ-12 >2 and WHODAS >16) will be randomised using computerized software on a 1:1 basis by an independent researcher not involved in the conduct of the study. The assessment team will record demographic information and will administer the Psychological Outcome Profiles instrument (PSYCHLOPS), Hospital Anxiety and Depression Scale (HADS), Patient Health Questionnaire (PHQ-9), PTSD Checklist Civilian version (PCL-C), Life Events Checklist for Pakistan (LECFP), Harvard Trauma Questionnaire (HTQ) events list, Multidimensional Scale of Perceived Social Support (MSPSS) and the Client Service Receipt Inventory (CSRI).

The post-intervention assessment (WHODAS, HADS, PHQ-9, PCL-C, MSPSS and PSYCHLOPS) will be scheduled at 7 weeks after the pre-intervention assessment (i.e. 1 week after the 5th PM+ session), and the follow-up assessment (WHODAS, HADS, PHQ-9, PCL-C, PSYCHLOPS, MSPSS and CSRI) will be scheduled at 13 weeks after the post-intervention assessment (i.e. 20 weeks after inclusion, in line with the timing of the follow-up assessment for the PM+ plus TAU participants). Table 1 presents an overview of measures that are administered at each of the assessments.

**Table 1:** Overview of instruments and assessments

Concept	Pre-assessment measures	Post-treatment assessment measures	3-months post-treatment follow-up assessment measures
Psychological distress	GHQ-12 (screener)		
Functioning	WHODAS (screener, primary outcome)	WHODAS	WHODAS
Mental disorders	HADS (primary outcome)	HADS (primary outcome)	HADS (primary outcome)
	PCL-C	PCL-C	PCL-C
	PHQ-9	PHQ-9	PHQ-9
Perceived problems	PSYCHLOPS	PSYCHLOPS	PSYCHLOPS
Adverse life events	HTQ events list LELFP		
Costs of care	CSRI		CSRI

Ongoing supervision of assessors will be conducted by the study coordinator. The assessment team will be blind to the allocation status of the participants.

#### Sample size and power calculations

Sample size calculation is based on a multicentre study of culturally-adapted cognitive behavioural therapy (CBT)-based intervention conducted in Pakistan that used HADS as the primary outcome measure [22]. A two-point reduction in HADS depression score between the intervention and control group is considered to be clinically relevant. With *p* < .05 and 90% power, a total of 96 participants are needed. Accounting for an expected drop-out rate of 50%, the total sample size is 192 participants, who will be equally randomized to PM+ plus TAU (n = 96) and TAU only (n = 96).

#### The Problem Management Plus (PM+) program

Developed by the WHO, PM+ seeks to ameliorate symptoms of common mental health problems [23, 24]. PM+ is delivered over 5 weekly sessions with 90 min duration for each. The PM+ program is being made available in different formats, however, in the current RCT, the PM+ individual version will be tested.

Session one orients participants to the program with motivational interviewing techniques to improve engagement, provides psychoeducation about common reactions to adversity, and teaches participants a basic stress management strategy. The latter strategy is practiced at the conclusion of every subsequent session to enhance learning. Session two addresses a participant-selected problem through the provision of problem solving techniques and introduces commencement of behavioural activation procedures. Sessions three and four continue to support participants’ application of problem solving, behavioural activation, and relaxation exercises, and introduces strategies to strengthen social support networks. In session five, education about retaining treatment gains is given, all learned strategies are reviewed, and the program is finished.

Problem Management Plus providers will be trained therapists, who have received eight days training in PM+ by the master trainer. All the PM+ providers in this trial had a master’s degree (16 years of education) in psychology. The adherence of the PM+ protocol will be assured by completing a self-check for each session by the PM+ providers, supported by peer-supervision. Fidelity of intervention will be ensured by fortnightly supervisions with the master trainer through Skype.

#### Treatment-as-Usual (TAU)

Treatment-as-Usual in the outpatients department for individuals with common mental disorders usually consists of an initial evaluation by trainee psychologists and psychiatrists followed by an expert consultation on the case. The main stay of treatment at specialized mental health care facilities is pharmacotherapy. Counselling and psychotherapy are frequently advised but lack of standardized training in psychotherapies and evidence base packages of care make the counselling non-specific and non-replicable. We will record complete details of the treatments accessed by the trial participants from both arms by using the adapted Client Services Receipt Inventory [25] at baseline and follow-up.

#### Screening measures

GHQ-12 [18, 19] assesses level of general psychological distress during screening. It consists of 12 questions that are scored on a four-point Likert scale ranging from 0 to 3. When used as a screening tool, the GHQ-12 is usually scored bi-modally (i.e.-0,1), and the total score ranges between 0–12, with higher scores representing higher levels of distress. In previous studies in Pakistan, cut-offs of 1 or higher and 2 or higher have been reported and used for determining clinical caseness of common mental health disorders [19, 26, 27].

WHODAS [20] is a generic assessment instrument assessing health and disability. Simple to administer, it is applicable across all health states, including mental disorders, and across cultures. The WHODAS assesses difficulties people have due to their illness across six domains of functioning (cognition, mobility, self-care, getting along, life activities, and participation). Difficulties are scored over the last 30 days on a five-point Likert scale as none, mild, moderate, severe, or extreme. The 12-item interviewer administered version translated in Urdu will be used in this study as a screener as well as one of the primary outcome measures.

#### Primary outcomes

Primary outcomes are (a) states of anxiety and depression measured using the HADS [28, 29]. The HADS is a well-established 14-item scale consisting of two sub-scales: HADS-A (anxiety, seven items, range 0–21) and HADS-D (depression, seven items, range 0–21). Higher scores indicate more anxiety and/or depression. The Urdu version of the HADS showed satisfactory reliability and validity [29] (b) Functional impairment assessed using the WHODAS (described above).

#### Secondary outcomes

The PHQ-9 is nine-item instrument measuring presence and severity of depression during the past 2 weeks [30]. The PHQ-9 questions are derived from the 16-item version. Participants rate their responses on a four-point Likert scale ranging from “not at all” to “nearly every day”. The PHQ-9 total severity score ranges from 0 to 27. The PHQ has been validated in Urdu [31, 32].

DSM IV posttraumatic stress disorder (PTSD) symptoms during the past week will be measured using the 17-item PCL-C [33]. Items are rated on a 1–5 scale and add up to a total severity score of 85. The PCL-C has been used previously in Pakistan [34] and has been found to have acceptable psychometric properties (Mushtaq, unpublished data, 2013). The PCL-C will be adapted to ask for symptoms in the last week (rather than month) to enhance sensitivity to change.

PSYCHLOPS [35] assesses progress on problems for which the person seeks help. It consists of four questions that encompass three domains: problems (2 questions), functioning (1 question) and wellbeing (1 question). Participants are asked to give free text responses to the problem and function domains. Responses are scored on an ordinal six-point scale producing a maximum score of 20 (6 points per domain). The PSYCHLOPs version administered at post-treatment and follow-up also includes an overall evaluation question (determining self-rated outcome ranging from “much better” to “much worse”). PSYCHLOPS has been validated in primary care populations across several countries [36, 37].

The MSPSS [38, 39] aims to measure perceived social support. It includes 12 items which cover three dimensions: family, friends and significant others. Each item is rated on a seven-point Likert-scale (1 = very strongly disagree; 7 = very strongly agree). A total score is calculated by summing the results for all items (range 12–84) with higher scores indicating higher levels of perceived social support. The MSPSS has been validated in Urdu [39].

#### Cost-effectiveness measures

Indicators of economic impact will be assessed using the WHODAS question on days out of role and the CSRI. The CSRI was developed for the collection of data on service utilization and related characteristics of people with mental disorders, as the basis for calculating the costs of care for mental health cost-effectiveness research [25]. It has been previously used in Pakistan and India [40, 41].

#### Other measures

Data on socio-demographic information including sex, age, education, marital status and work status) will be collected.

To assess the experience of potentially traumatic events, part one of the HTQ [42] will be administered. This includes 17 items describing a range of traumatic events, such as: “lack of food and water”, “forced separation from family members”, and “being close to death”. Each event is rated as either present (1) or absent (0). The HTQ has been validated and applied in many countries, including Pakistan [43].

Life events other than potentially traumatic events (e.g. loss of job, housing problems, financial difficulties, problems with the law, marital problems, bereavement, etc.) are assessed using a life events measure previously developed for the Pakistani population (LECFP [44]). Life events are rated as either present (1) or absent (0).

#### Process evaluation

The feasibility, difficulties and successes in carrying out intervention activities will be explored through comprehensive process monitoring and semi-structured interviews with key stakeholders including PM+ providers, intervention recipients and clinical staff involved in the study.

Individual semi-structured interviews will be conducted within 4 weeks after the conduct of final outcome assessments with five participants from each category. The aim of these interviews is to explore their perceptions of the benefits and challenges of integrating PM+ into the routine service provision. Interviews will follow a semi-structured interview guide including following topics: overall impressions of PM+, experiences of PM training and supervision, rapport with participants and families of participants, views on the delivery format, experiences of participants’ intervention adherence and strategies to keep participants motivated, view on intervention scalability and integration into the existing systems.

Qualitative interviews and data analysis will be conducted by a pair of researchers independent of the research team to avoid any bias in responses. Researchers will be trained in qualitative interviewing and will be provided supervision throughout data collection and analysis process by a senior researcher. All the interviews will be recorded and transcribed. Analysis will be conducted manually following a framework analysis approach.

Process monitoring includes the review of PM+ providers’ records of sessions with participants, PM+ providers’ supervision records including intervention fidelity monitoring and supervision of supervisors by the master trainer. This data will be collected throughout the intervention delivery and reviewed as it is collected, leading to an iterative process of intervention monitoring informing intervention delivery.

Findings from this phase of the study will be used to inform the set-up of PM+ clinics at specialized mental health care facilities locally, nationally and internationally.

#### Analysis

Primary analyses will be based on intent-to-treat population and secondary analyses will be based on per-protocol population.

The primary outcome will be summarised using number of subjects (n), means, standard deviations (SD), minimum, and maximum.

To estimate the treatment effect, a linear mixed model will be employed for the primary endpoint analysis, which will have treatment, as fixed effects, baseline measurement of primary endpoint as covariate, and subject as random effects. The mean difference between two treatment arms at each visit/time together with its 95% confidence interval will be derived from the mixed model. Covariate-adjusted mixed model of primary endpoint will also be performed by adding pre-specified covariates at baseline into the above model.

The details of the analysis can be found in the attached Statistical Analysis Plan (SAP) (Additional file 2: Appendix B).

##### Cost-effectiveness analysis

We will also analyse aggregated health care costs, computed from costs of treatment (primary care, outpatient hospital visits, inpatient admission, diagnostic tests and investigations, drug prescriptions), and the aggregated patient and family costs (number of days with reduced working hours, informal caregiving time by relatives or friends), and travel costs and time spent travelling to or waiting for consultations. The between-group comparison of mean costs will be completed using a standard *t* test with ordinary least squares regression used for adjusted analyses, with the validity of results confirmed using bootstrapping.

Analyses of the data will be carried out in SPSS version 21 and SAS 9.3. Across all analyses, two-tailed tests will be reported with *p* < .05.

#### Adverse events reporting

All adverse events (AEs) and serious adverse events (SAEs) reported spontaneously by the subject or observed by the investigators or other staff members will be recorded by the research team. We consider an event a SAE if it is an undesirable experience occurring to a subject during the study, whether or not considered related to the research procedure. Although it is unlikely that SAEs would occur given the nature of the intervention, all adverse events and SAEs will be reported to the local independent advisory board. The chair or a nominated person from the advisory board will review SAEs within 48 h and the advisory board will review all AEs twice a month and where necessary to determine any appropriate action in respect of ongoing trial conduct. On the informed consent form, information is included to inform participants that the research coordinator, independent assessor, or another clinician other than their PM+ provider are available to them if they are upset by this study. The principal investigator will inform the participants and the IRB if any adverse event takes place, on the basis of which it appears that the disadvantages of participation may be significantly greater than was foreseen in the research proposal.

## Discussion

Estimated deficit of trained health care workers globally is around 4 million; factors such as migration and brain drain phenomenon further aggravate the situation in low resource settings [45]. Task shifting is an established implementation strategy to bridge the treatment gap due to lack of specialist human resource in low resource settings. Task shifting strategies empower frontline workers such as nurses and non-specialist providers to perform specific roles to bridge the treatment gap, sustain and scale-up care for priority health conditions [46].

Primary health care and community based trials of psychological interventions in LMICs have furnished evidence for the effectiveness of task shifting approach in global mental health [10, 11, 17, 47–49]. This is a unique study to evaluate the effectiveness and cost effectiveness of integrating task shifting approach in the provision of evidence based psychological therapy in a specialised mental health care setting of Pakistan.

Specialized mental health care facilities are the main source of care for mental health in LMICs, however, availability of evidence based psychological interventions even in specialised mental health care facilities remain a challenge in such settings [6]. In many low resource settings, where there are not enough psychiatrists; trainee psychiatrists, psychologists, psychiatric nurses, social workers and community based voluntary agents play an important role in the provision of care for mental health. In many of these settings training opportunities in specialised, safe and effective psychological interventions are not always available. Training in a brief trans-diagnostic psychological intervention programme, endorsed by the WHO, such as PM+, is envisioned to increase the provision of safe and effective care for mental health by non-specialist under the supervision of specialists to bridge the treatment gap for mental health care in low resource settings.

Evaluating the implementation, effectiveness and cost-effectiveness of integrating PM+ in routine care services will generate evidence to improve access of psychological interventions in low resource settings and will serve as a model of integrating non-specialist delivered, evidence based, cost-effective psychological interventions for common metal disorders in specialized healthcare settings globally.

## Trial status

At the time of manuscript submission, trial was ongoing. Results of this study are expected early 2017.

## Additional files

**Additional file 1.** Appendix A.

**Additional file 2.** Appendix B.

## Abbreviations

AE: adverse event
CBT: cognitive behavioural therapy
CSRI: Client Service Receipt Inventory
GHQ-12: General Health Questionnaire-12
HADS: Hospital Anxiety and Depression Scale
HDRF: Human Development Research Foundation
HTQ: Harvard Trauma Questionnaire
LECFP: Life Events Checklist for Pakistan
LMIC: low and middle income country
mhGAP: mental health Gap Action Program
MSPSS: Multidimensional Scale of Perceived Social Support
PCL-C: PTSD Checklist Civilian verson
PHQ-9: Patient Health Questionnaire
PM+: Problem Management Plus
PSYCHLOPS: Psychological Outcome Profiles instrument
PTSD: posttraumatic stress disorder
RCT: randomised controlled trial
SAE: serious adverse event
SAP: statistical analysis plan
TAU: Treatment-as-Usual
WHO: World Health Organization
WHO-AIMS: World Health Organization Assessment Instrument for Mental Health Systems
WHODAS: WHO Disability Assessment Schedule
