# Correction to: Problem Management Plus (PM+) in the management of common mental disorders in a specialized mental healthcare facility in Pakistan; study protocol for a randomized controlled trial

**DOI:** 10.1186/s13033-018-0231-1

**Published:** 2018-09-27

**Authors:** Syed Usman Hamdani, Zainab Ahmed, Marit Sijbrandij, Huma Nazir, Aqsa Masood, Parveen Akhtar, Hania Amin, Richard A. Bryant, Katie Dawson, Mark van Ommeren, Atif Rahman, Fareed Aslam Minhas

**Affiliations:** 1 Institute of Psychiatry, WHO Collaborating Centre for Mental Health Research and Training, Benazir Bhutto Hospital, Rawalpindi, Pakistan; 2 Human Development Research Foundation, Islamabad, Pakistan; 3 Department of Clinical Psychology, VU University, Amsterdam, The Netherlands; 4 School of Psychology, University of New South Wales, Sydney, Australia; 5 World Health Organization (WHO), Department of Mental Health and Substance Abuse, Geneva, Switzerland; 6 University of Liverpool, Liverpool, UK

## Correction to: Int J Ment Health Syst (2017) 11:40 10.1186/s13033-017-0147-1

In the original version of this article [1], the reference 35 was published incorrectly. The corrected reference is given below:

Ashworth M, Robinson S, Godfrey E, Shepherd M, Evans C, Seed P, Parmentier H, Tylee A. Measuring mental health outcomes in primary care: the psychometric properties of a new patient-generated outcome measure, PSYCHLOPS (Psychological Outcome Profiles). Prim Care Ment Health. 2005;3:261–270.
