# Supplementary material for: Problem Management Plus (PM+) in the management of common mental disorders in a specialized mental healthcare facility in Pakistan; study protocol for a randomized controlled trial
Source: Int J Ment Health Syst. 2017 Jun 8;11:40. doi: 10.1186/s13033-017-0147-1 (PMC5465445; doi:10.1186/s13033-017-0147-1)
Supplement: Supplementary file 2 — Additional file 2. Appendix B. [file 13033_2017_147_MOESM2_ESM.docx]

**Statistical Analysis Plan**

Effectiveness and cost-effectiveness evaluation of PM+ plus Treatment as Usual (TAU) versus Treatment as Usual (TAU) in the management of common mental disorders in a tertiary mental healthcare facility in Pakistan: a single blind Randomized Control Trial (RCT)

**V 3.0**

**October, 2016**

**Table of Contents**

[1. INTRODUCTION 1](#_Toc464294739)

[2. STUDY OBJECTIVES AND OUTCOMES 1](#_Toc464294740)

[2.1. Primary Objective 1](#_Toc464294741)

[2.2. Secondary Objectives 1](#_Toc464294742)

[3. STUDY DESIGN 2](#_Toc464294743)

[3.1. Design 2](#_Toc464294744)

[3.2. Interventions 2](#_Toc464294745)

[3.3. Randomisation 2](#_Toc464294746)

[3.4. Sample Size 2](#_Toc464294747)

[3.5. Masking 2](#_Toc464294748)

[4. ANALYSIS POPULATIONS 3](#_Toc464294749)

[4.1. Population Data Sets 3](#_Toc464294750)

[4.2. Analysis Close Date 3](#_Toc464294751)

[4.3. Data Management 3](#_Toc464294752)

[4.4. Data Cleaning 3](#_Toc464294753)

[4.5. Data Download 3](#_Toc464294754)

[5. EFFECTIVENESS ANALYSES 3](#_Toc464294755)

[5.1. Primary Outcome Analysis 3](#_Toc464294756)

[5.1.1. ITT analysis of primary outcome 3](#_Toc464294757)

[5.1.2. PP analysis of primary outcome 4](#_Toc464294758)

[5.1.3. Sensitivity analyses of primary outcome 4](#_Toc464294759)

[5.2. Secondary Outcome Analysis 4](#_Toc464294760)

[5.2.1. Analysis of secondary economic outcome 4](#_Toc464294761)

[5.2.2. Analysis of secondary outcomes with repeated measurements 4](#_Toc464294762)

[5.2.3. Analysis of other secondary outcomes 5](#_Toc464294763)

[6. SAFETY ANALYSES 5](#_Toc464294764)

[6.1. Safety Variables 5](#_Toc464294765)

[6.2. Additional Safety Analyses 5](#_Toc464294766)

[7. GENERAL CONSIDERATION FOR DATA ANALYSES 5](#_Toc464294767)

[7.1. Covariates Analyses 5](#_Toc464294768)

[7.2. Subgroup Analysis 5](#_Toc464294769)

[7.3. Multiplicity 5](#_Toc464294770)

[7.4. Other Data Considerations 5](#_Toc464294771)

[7.4.1. Data Summaries 5](#_Toc464294772)

[7.4.2. Graphical Displays 5](#_Toc464294773)

[8. PARTICIPANT FLOW CHART 6](#_Toc464294774)

[Follow-Up 6](file:///E:\Parveen%20PC\HDRF\PM%20+%20clinic%20RCT\Protocol%20paper\PM+%20IOP%20_SAP_V2_110816.docx#_Toc464294775)

[Analysis 6](file:///E:\Parveen%20PC\HDRF\PM%20+%20clinic%20RCT\Protocol%20paper\PM+%20IOP%20_SAP_V2_110816.docx#_Toc464294776)

[Enrollment 6](file:///E:\Parveen%20PC\HDRF\PM%20+%20clinic%20RCT\Protocol%20paper\PM+%20IOP%20_SAP_V2_110816.docx#_Toc464294777)

[Allocation 6](file:///E:\Parveen%20PC\HDRF\PM%20+%20clinic%20RCT\Protocol%20paper\PM+%20IOP%20_SAP_V2_110816.docx#_Toc464294778)

[9. REFERENCES 8](#_Toc464294779)

**ABBREVIATIONS**

| **Abbreviation** | **Explanation** |
| --- | --- |
| CSRI | Client Service Receipt Inventory |
| TAU | Treatment as usual |
| HADS | Hospital Anxiety and Depression Scale |
| HADS-A | Hospital Anxiety and Depression Scale-Anxiety |
| HADS-D | Hospital Anxiety and Depression Scale-Depression |
| HTQ | Harvard Trauma Questionnaire |
| MSPSS | Multidimensional Scale of Perceived Social Support |
| PCL-4 | Post-Traumatic Stress Disorder Checklist |
| PHQ-9 | Patient Health Questionnaire |
| PM+ | Problem Management Plus |
| PSYCHLOPS | Psychological Outcomes Profile Instrument |
| PTSD | Post-Traumatic Stress Disorder |
| SAP | Statistical Analysis Plan |

# 1. INTRODUCTION

The purpose of this Statistical Analysis Plan (SAP) is to define the effectiveness, economic and qualitative evaluation variables, statistical methods, and analysis strategies to deal with objectives of the study in evaluating effectiveness and cost-effectiveness of PM+ plus Treatment as Usual (TAU) versus Treatment as Usual (TAU) in the management of common mental disorders in a tertiary mental healthcare facility in Pakistan through a randomised controlled trial.

# 2. STUDY OBJECTIVES AND OUTCOMES

## 2.1. Primary Objective

To determine whether a newly developed Problem Management Plus (PM+) intervention is effective compared to a control group in reducing symptoms of psychological distress (anxiety and depression) and improve the levels of functioning in individuals attending a specialist mental health care facility in Rawalpindi, Pakistan.

**Primary Outcome:** a. change in scores of depression and anxiety measured by Hospital Anxiety and Depression Scale (HADS) [1] at 20-week b). Change in scores of functional disability measured by WHODAS [1] at 20-week.

## 2.2. Secondary Objectives

To assess post-traumatic stress symptoms, perceived social support, and cost-effectiveness.

**Secondary Clinical Outcomes:**

1. The combined HADS, HADS-A, and HADS-D measured at baseline, 7-week, and 20-week.
2. Depressive disorder measured by the Primary Health Questionnaire (PHQ-9) [3] , social support measured by the Multidimensional Scale of Perceived Social Support (MSPSS) [4] , self-reported wellbeing by the Psychological Outcomes Profile Instrument (PSYCHLOPS) [5] and post-traumatic stress disorder (PTSD) symptoms measured by the post-traumatic stress disorder checklist (PCL), [6] at baseline, 7-week, and 20-week, respectively.

**Secondary Economic Outcome:**

Economic effectiveness on cost of care is measured using the Client Service Receipt Inventory (CSRI) [7] at baseline and 20-week.

**Secondary qualitative evaluation outcome:**

Up to 6 semi-structured interviews for assessing intervention acceptability are provided for a random sub-sample including intervention facilitators, intervention participants with equal numbers of completers and drop-outs, control arm participants, physicians /psychiatrists research assistants, and family members of intervention participants. A semi-structured topic guide regarding topics relevant to each category of respondent is followed by the interview.

**Additional Safety Variables:**

No safety data are collected in this study.

**Primary Analytical Subset:**

Primary analyses will be based on intent-to-treat population and secondary analyses will be based on per-protocol population.

# 3. STUDY DESIGN

## 3.1. Design

This study is a prospective, single-blinded, individually randomised controlled trial with two parallel arms and equal randomisation of eligible patients to a PM+ group with 5 sessions of manualised comprehensive intervention and to a treatment as usual group (TAU).

## 3.2. Interventions

At baseline, after completion of all assessments but before randomisation, a standard, simple explanation of the nature of common mental disorders will be given to all participants.

**Intervention group:** The manualised PM+ intervention involves empirically supported strategies of problem-solving, behavioural activation, accessing social support, and stress management training [^[[1]](#endnote-1)^] Prior to implementation, a period of formative work was undertaken to contextually adapt the protocol for delivery in, Pakistan.

**Control group:** Following randomisation, the control group participants will only receive routine care from the tertiary mental healthcare facility

Both of the two group participants will continue to receive routine care from the tertiary health care facility on an individual basis.

## 3.3. Randomisation

192 participants will be randomised to the intervention and control arms on a 1:1 allocation using simple randomisation method. Random allocation software is used to generate a randomisation list by an independent statistician.

## 3.4. Sample Size

Sample size calculation is based on a multi-center study of culturally adapted CBT based intervention conducted in Pakistan that used HADS as the primary outcome measure [8]. A two point reduction in HADS depression score between the intervention and control group has been considered to be clinically significant. With 5% significance level and 90% power it was calculated that a total of 96 participants are needed. With a 50% expected drop-out rate, the total sample size estimated to be 192 equally randomized to intervention and control arms

## 3.5. Masking

Given the nature of PM+ intervention, it is not possible to mask the participants and facilitators, as well as the qualitative research team staffs. All researchers conducting outcome assessments will be masked in the trial. The trial statistician will also be blinded regarding the treatment code when he develops the statistical analysis plan and writes the statistical programmes, which will be validated and completed using dummy randomisation codes. The actual allocation will only be provided to the study team after lock of the database.

# 4. ANALYSIS POPULATIONS

## 4.1. Population Data Sets

Two populations will be considered in the analysis as follows:

**Intent-to-Treat population**

Intent-to-treat (ITT) will be defined at the moment the randomisation is performed. For the primary outcome analysis in this trial, patients will be followed with their ITT arm. In analysis referring to a specific number of days, the randomisation day will be considered day 0.

**Per-Protocol Population**

Per-protocol (PP) population is based on the treatment actually received. This population will be used for the supportive analyse.

## 4.2. Analysis Close Date

The analysis close date is the date on which the last patient completed 20-week follow-up.

## 4.3. Data Management

**Quantitative data:** These data will be completed on paper assessment booklets with assigned participant codes, stored at the field office at the end of each day.

**Qualitative data:** These data will be stored in paper format in locked filling cabinets in the field office at the end of each day. None of the qualitative data will contain personal identified information (i.e., name, age, category of respondent, etc.).

**All other process data:** These data will be stored in locked filing cabinets in the field office. Intervention team members have been trained in the importance of de-identifying all notes relating to participants progress through the intervention to ensure confidentiality.

## 4.4. Data Cleaning

Data daily checking will be performed by the research coordinator, with queries identified and resolved promptly. Data will be doubly entered at the Human Development Research Foundation in Islamabad by an assigned data entry team. Discrepancies will be resolved by a third data entry person. The data will be checked to ensure that there are no erroneous entries and that all missing data is properly coded. Any changes will be made on the database.

## 4.5. Data Download

For each time point, once all data have been inputted and checked, the database will be password protected, with data managers controlling access to the passwords and database backed-up daily. The data will be download into SAS and SPSS formats for statistical analysis.

# 5. EFFECTIVENESS ANALYSES

## 5.1. Primary Outcome Analysis

### 5.1.1. ITT analysis of primary outcome

The primary outcome will be summarised using number of subjects (n), means, standard deviations (SD), minimum, and maximum.

To estimate the treatment effect, a linear mixed model will be employed for the primary endpoint analysis, which will have treatment as fixed effects, baseline measurement of primary endpoint as covariate, and subject as random effects. The mean difference between two treatment arms at each visit/time together with its 95% confidence interval will be derived from the mixed model. Covariate-adjusted mixed model of primary endpoint will also be performed by adding pre-specified covariates at baseline into the above model.

### 5.1.2. PP analysis of primary outcome

The main conclusion in the clinical report will be based on the ITT analysis of the primary outcome. A secondary analysis of the primary outcome will also be presented using the PP population.

### 5.1.3. Sensitivity analyses of primary outcome

Sensitivity to missing values

Missing data will be treated as missing at random in the mixed model analysis and no imputation of primary endpoint will be made. To assess the sensitivity of the result to this assumption, the last observation carried forward strategy will be used to compute missing primary endpoints.

Sensitivity to baseline covariates

The observed treatment effect regarding the primary outcome may be confounded due to imbalances of some baseline characteristics of participants which are associated with the primary outcome. To control for such potential confounding factors, a subgroup analysis will be performed on the pre-specified covariates.

## 5.2. Secondary Outcome Analysis

### 5.2.1. Analysis of secondary economic outcome

Health economic analysis will be conducted to determine the difference in costs and outcomes in the intervention arm as compared to the treatment as usual group.

Primary analysis will be a total costs over the 20-week follow-up treatment period. Between-group comparison of mean costs will be completed using standard *t*-test with ordinary least squares regression used for adjusted analyse, with the validity of results confirmed using bootstrapping [9].

Cost-effectiveness will be assessed by combining costs with the primary outcome measure in incremental cost-effectiveness analysis. Repeat re-sampling from the costs and effectiveness data (bootstrapping) will be used to calculate the probability that each of the treatments is the optional choice. A cost-effectiveness acceptability curve will be presented by plotting these probabilities for a range of possible values of the ceiling ratio [10]. The relationship between costs and the remaining outcome measures will be explored individually in a cost consequences analysis [10], presenting the relationship between costs and consequences without formal assessment for cost effectiveness.

### 5.2.2. Analysis of secondary outcomes with repeated measurements

The following five clinical outcomes are measured at baseline, 7-week and 20-week and treated as continuous variables: (1) anxiety and depression (using the HADS); (2) depression disorders (using the PHQ-9); (3) PTSD symptoms (using the PCL); (4) social support (using the MSPSS); and (5) self-reported wellbeing (using the PSYCHLOPS).

A linear mixed model mentioned in section 5.1.1 for the primary outcome analysis will be used for analysing the above outcomes.

### 5.2.3. Analysis of other secondary outcomes

Continuous secondary outcomes will be analysed in the similar way as the primary endpoint analysis. For the analysis of binary outcomes, generalised mixed model will be employed with treatment as fixed effects, baseline measurement as covariate, and subject as random effect. The odds ratio between two treatment arms at each visit together with its 95% confidence interval will be derived from the generalised mixed model.

Changes in caseness of depression will be calculated for the treatment completer sample using the recommended cut-off of 10 on the PHQ-9 and will analysed using a hierarchical logistic model with the same fixed and random effects as HLM models above, from which odds ratio of having a depression together with 95%CI at each time point will be derived.

# 6. SAFETY ANALYSES

## 6.1. Safety Variables

There are no measurements on safety data such as vital signs, ECG, blood laboratory data.

## 6.2. Additional Safety Analyses

There will be no additional safety analysis.

# 7. GENERAL CONSIDERATION FOR DATA ANALYSES

## 7.1. Covariates Analyses

Covariate analyses will be performed on the primary outcome (See Section 5.1.3).

## 7.2. Subgroup Analysis

Subgroup analyses will be performed for all outcome variables.

## 7.3. Multiplicity

Multiplicity adjustment will be apply to the primary and secondary outcome analyses.

## 7.4. Other Data Considerations

### 7.4.1. Data Summaries

Continuous variables will be summarised according to number of subjects with non-missing data (n), means, standard deviations (SD), median, minimum, and maximum. The confidence interval (CI) will be added on summaries of continuous effectiveness variables.

Categorical variables will be summarised according to the absolute frequency and percentage of subjects (%) in each category level. The denominator for the percentages is the number of subjects in the treatment arm with data available, unless noted otherwise.

### 7.4.2. Graphical Displays

Mean scores for primary and some secondary outcomes by treatment and visit will be plotted.

# 8. PARTICIPANT FLOW CHART


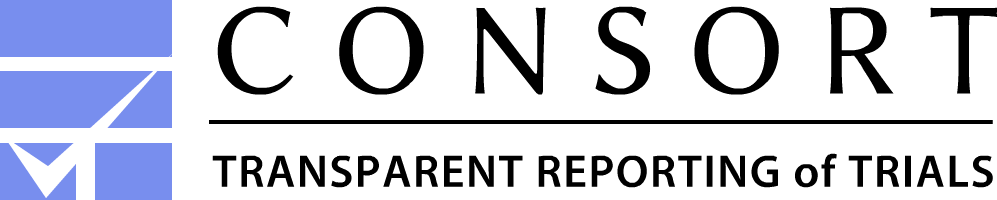


**CONSORT 2010 Flow Diagram**

## Follow-Up

Analysed (n= )
♦ Excluded from analysis (give reasons) (n= )

## Analysis

Analysed (n= )
♦ Excluded from analysis (give reasons) (n= )

Lost to follow-up (give reasons) (n= )

Discontinued intervention (give reasons) (n= )

Lost to follow-up (give reasons) (n= )

Discontinued intervention (give reasons) (n= )

## Enrollment

Allocated to intervention (n= )

♦ Received allocated intervention (n= )

♦ Did not receive allocated intervention (give reasons) (n= )

## Allocation

Allocated to intervention (n= )

♦ Received allocated intervention (n= )

♦ Did not receive allocated intervention (give reasons) (n= )

Randomized (n= )

Excluded (n= )

♦  Not meeting inclusion criteria (n= )

♦  Declined to participate (n= )

♦  Other reasons (n= )

Assessed for eligibility (n= )

| **Variables (instrument)** | **Measurement time point** | | |
| --- | --- | --- | --- |
|  | **Baseline** | **7-week** | **20-week** |
| Anxiety and depression (HADS) | √ | √ | √ |
| WHODAS | √ | √ | √ |
| Depressive disorders (PHQ-9) | √ | √ | √ |
| Adverse life events (Life Events Checklist for Pakistan; Events Section of HTQ) | √ |  |  |
| Post-traumatic stress disorder checklist (PCL) | √ | √ | √ |
| Perceived social support (MSPSS) | √ | √ | √ |
| Self-reported wellbeing (PSYCHLOPS) | √ | √ | √ |
| Cost of care (CSRI) | √ |  | √ |

#

# 9. REFERENCES

1. 1. Mumford DB, Tareen IA, Bajwa MA, Bhatti MR, Karim R: **The translation and evaluation of an Urdu version of the Hospital Anxiety and Depression Scale.** *Acta Psychiatr Scand* 1991, **83:**81-85.
   2. WHO: *Measuring health and disability; Manual for WHO Disability Assessment Schedule WHODAS 2.0.* Geneva, Switzerland: WHO; 2010.
   3. Husain N, Gater R,3. Tomenson B, Creed F: **Comparison of the Personal Health Questionnaire and the Self Reporting Questionnaire in rural Pakistan.** *J Pak Med Assoc* 2006, **56:**366-370.
   4. Akhtar A, Rahman A, Husain M, Chaudhry IB, Duddu V, Husain N: **Multidimensional scale of perceived social support: psychometric properties in a South Asian population.** *J Obstet Gynaecol Res* 2010, **36:**845-851.
   5. Ashworth MS, M.; Christey, J.; Matthews, V.; Wright, K.; Parmentier, H.; Robinson, S.; Godrey, E.: **A client-generated psychometric instrument: the development of "PSYCHLOPS".** *Counselling and Psychotherapy Research: Linking Research with Practice* 2013, **4:**27-31.
   6. Weathers FWL, B.T.; Huska,J.A.; Keane, T.M.;: *The PTSD checklist—civilian version.* Scale available at from the National Center for PTSD at [www.ptsd.va.gov](http://www.ptsd.va.gov) 1994.
   7. Buttorff C, Hock RS, Weiss HA, Naik S, Araya R, Kirkwood BR, Chisholm D, Patel V: **Economic evaluation of a task-shifting intervention for common mental disorders in India.** *Bull World Health Organ* 2012, **90:**813-821
   8. Naeem F, Gul M, Irfan M, Munshi T, Asif A, Rashid S, Khan MN, Ghani S, Malik A, Aslam M, et al: **Brief Culturally adapted CBT (CaCBT) for depression: A randomized controlled trial from Pakistan.** *J Affect Disord* 2015, **177:**101-107.
   9. Linder D., F. & Rempala. G., A. (2015). **Bootstrapping least-squares estimates in biochemical reaction networks.** Journal of biological dynamics, 9(1), 125-146.
   10. Araki, D., & Kamae, I. (2015). **The Augmented Representation of the Cost-effectiveness Acceptability Curve for Economic Evaluation of Health Technology**. The Kobe journal of the medical sciences, 61(1), 9-18.

   [↑](#endnote-ref-1)
